# *tert*-Butyl *N*-hydr­oxy-*N*-[(1S*,2R*)-2-(1-naphth­yl)cyclo­pent-3-en-1-yl]carbamate

**DOI:** 10.1107/S1600536809020315

**Published:** 2009-06-06

**Authors:** Alan J. Lough, Ben P. Machin, William Tam

**Affiliations:** a Department of Chemistry, University of Toronto, Toronto, Ontario, Canada M5S 3H6; b Department of Chemistry, University of Guelph, Guelph, Ontario, Canada N1G 2W1

## Abstract

The relative stereochemistry of the title compound, C_20_H_23_NO_3_, was established by X-ray analysis. The asymmetric unit contains two independent mol­ecules. In the crystal structure, each type of mol­ecule forms a centrosymmetric dimer *via* pairs of inter­molecular O—H⋯O hydrogen bonds, resulting in an *R*_2_^2^(10) loop in each case.

## Related literature

For hydrogen-bond graph sets, see: Bernstein *et al.* (1995 ▶).
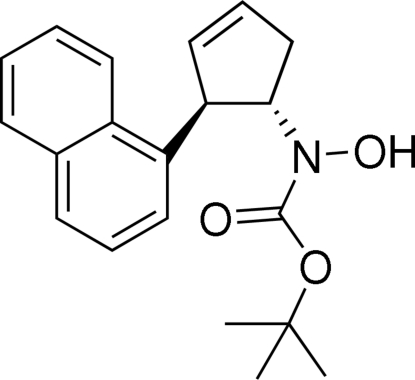


## Experimental

### 

#### Crystal data

C_20_H_23_NO_3_
*M* *_r_* = 325.39
Triclinic, 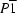

*a* = 8.4710 (5) Å
*b* = 8.4880 (4) Å
*c* = 26.1836 (12) Å
α = 95.980 (3)°
β = 95.419 (2)°
γ = 111.960 (2)°
*V* = 1718.32 (15) Å^3^
*Z* = 4
Mo *K*α radiation
μ = 0.08 mm^−1^
*T* = 150 K
0.22 × 0.18 × 0.14 mm

#### Data collection

Nonius KappaCCD diffractometer
Absorption correction: multi-scan (*SORTAV*; Blessing, 1995 ▶) *T*_min_ = 0.918, *T*_max_ = 0.989
10769 measured reflections
6622 independent reflections
4089 reflections with *I* > 2σ(*I*)
*R*_int_ = 0.035

#### Refinement

*R*[*F* ^2^ > 2σ(*F* ^2^)] = 0.057
*wR*(*F* ^2^) = 0.167
*S* = 1.10
6622 reflections
448 parameters
H atoms treated by a mixture of independent and constrained refinement
Δρ_max_ = 0.33 e Å^−3^
Δρ_min_ = −0.25 e Å^−3^

### 

Data collection: *COLLECT* (Nonius, 2002 ▶); cell refinement: *DENZO-SMN* (Otwinowski & Minor, 1997 ▶); data reduction: *DENZO-SMN*; program(s) used to solve structure: *SIR92* (Altomare *et al*., 1994 ▶); program(s) used to refine structure: *SHELXTL* (Sheldrick, 2008 ▶); molecular graphics: *PLATON* (Spek, 2009 ▶); software used to prepare material for publication: *SHELXTL*.

## Supplementary Material

Crystal structure: contains datablocks global, I. DOI: 10.1107/S1600536809020315/hb2980sup1.cif

Structure factors: contains datablocks I. DOI: 10.1107/S1600536809020315/hb2980Isup2.hkl

Additional supplementary materials:  crystallographic information; 3D view; checkCIF report

## Figures and Tables

**Table 1:** Hydrogen-bond geometry (Å, °)

*D*—H⋯*A*	*D*—H	H⋯*A*	*D*⋯*A*	*D*—H⋯*A*
O1*A*—H1*OA*⋯O3*A*^i^	0.96 (4)	1.75 (4)	2.689 (3)	165 (3)
O1*B*—H1*OB*⋯O3*B*^ii^	0.88 (3)	1.84 (4)	2.714 (3)	179 (4)

Symmetry codes: (i) 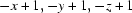; (ii) 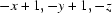.

**Fig. 1.**
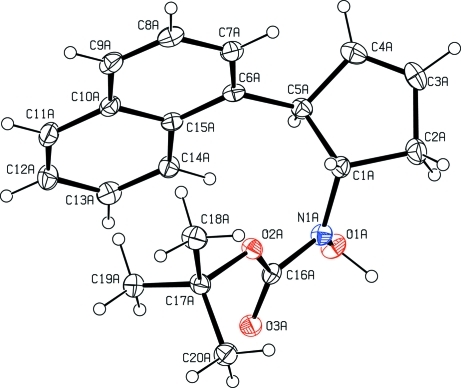
View of molecule A of (I): displacement ellipsoids are drawn at the 30% probabilty level.

**Fig. 2.**
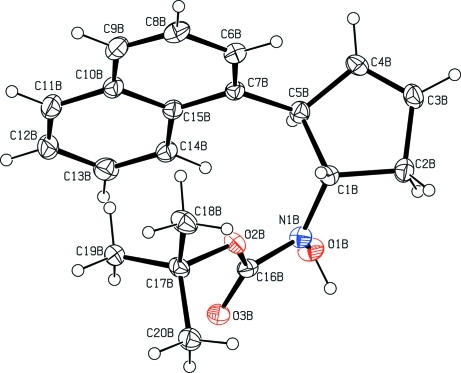
View of molecule B of (I): displacement ellipsoids are drawn at the 30% probabilty level.

**Fig. 3.**
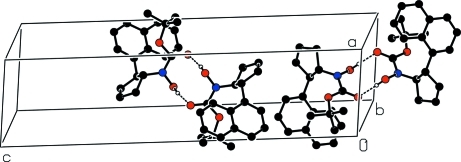
Part of the crystal structure with hydrogen bonds shown as dashed lines.

**Fig. 4.**
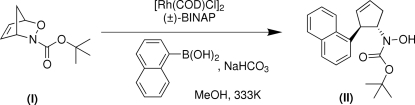
The synthetic scheme

## supplementary crystallographic information

### Comment

We have recently studied the addition of aryl groups to a 3-aza-2-oxabicyclo[2.2.1]hept-5-ene system using a [Rh(COD)Cl]_2_ (COD = cyclooctadiene) catalyst. The reaction produces two stereoisomers, the absolute stereochemistry of the title major isomer,(I), was determined by single-crystal X-ray diffraction.

The asymmetric unit of (I) contains two independent molecules [A and B] which are shown in Figs 1 and 2. In the crystal structure, each type of molecule is linked into a centrosymmetric dimer *via* intermolecular O—H···O hydrogen bonds (Fig. 3). Each dimer forms a *R*_2_^2^(10) graph set (Bernstein *et al.*, 1995).

### Experimental

3-aza-2-oxabicyclo[2.2.1]hept-5-ene (I) (see Fig. 4) (99.4 mg, 0.504 mmol) and 1-napthalene boronic acid (103.1 mg, 0.599 mmol) were weighed into a dry vial and purged with nitrogen. Dried MeOH (2.3 ml) was measured out into a dry vial and purged with nitrogen. Inside an inert atmosphere (Ar) dry box, [Rh(COD)Cl]_2_ (15.6 mg, 0.031 mmol) and (±)-BINAP (41.0 mg, 0.066 mmol) were weighed out and dissolved in methanol (1.0 ml), and stirred for 30 minutes. NaHCO_3_ (85.4 mg, 1.03 mmol) was added to the vial containing the bicyclic alkene and dissolved in MeOH (1.3 ml) and transferred to the vial with the catalyst. The reaction was heated to 333 K and stirred overnight. The crude product was purified using column chromatography (EtOAc:hexanes = 1:4) to give (II) as the major stereoisomer as an off white solid (58.1 mg, 0.179 mmol, 36%).  Colourless blocks of (I) were grown from a solution of the title compound in dichloromethane/hexanes.

### Refinement

H atoms bonded to C atoms were placed in calculated positions with C—H = 0.95–1.00 and they were included in the refinement in a riding-model approximation with *U*_iso_(H) = 1.2*U*_eq_(C) or 1.5*U*_eq_(C) for methyl C atoms. H atoms bonded to O atoms were located in difference maps and refined independently with isotropic displacement parameters.

### Crystal data

**Table:**

C_20_H_23_NO_3_	*Z* = 4
*M**_r_* = 325.39	*F*(000) = 696
Triclinic, *P*1	*D*_x_ = 1.258 Mg m^−^^3^
Hall symbol: -P 1	Mo *K*α radiation, λ = 0.71073 Å
*a* = 8.4710 (5) Å	Cell parameters from 10769 reflections
*b* = 8.4880 (4) Å	θ = 2.6–26.3°
*c* = 26.1836 (12) Å	µ = 0.08 mm^−^^1^
α = 95.980 (3)°	*T* = 150 K
β = 95.419 (2)°	Block, colourless
γ = 111.960 (2)°	0.22 × 0.18 × 0.14 mm
*V* = 1718.32 (15) Å^3^	

### Data collection

**Table:**

Nonius KappaCCD diffractometer	6622 independent reflections
Radiation source: fine-focus sealed tube	4089 reflections with *I* > 2σ(*I*)
graphite	*R*_int_ = 0.035
Detector resolution: 9 pixels mm^-1^	θ_max_ = 26.3°, θ_min_ = 2.6°
φ scans and ω scans with κ offsets	*h* = −10→10
Absorption correction: multi-scan (*SORTAV*; Blessing, 1995)	*k* = −10→10
*T*_min_ = 0.918, *T*_max_ = 0.989	*l* = −32→30
10769 measured reflections	

### Refinement

**Table:**

Refinement on *F*^2^	Secondary atom site location: difference Fourier map
Least-squares matrix: full	Hydrogen site location: inferred from neighbouring sites
*R*[*F*^2^ > 2σ(*F*^2^)] = 0.057	H atoms treated by a mixture of independent and constrained refinement
*wR*(*F*^2^) = 0.167	*w* = 1/[σ^2^(*F*_o_^2^) + (0.0652*P*)^2^ + 0.4209*P*] where *P* = (*F*_o_^2^ + 2*F*_c_^2^)/3
*S* = 1.10	(Δ/σ)_max_ < 0.001
6622 reflections	Δρ_max_ = 0.33 e Å^−^^3^
448 parameters	Δρ_min_ = −0.24 e Å^−^^3^
0 restraints	Extinction correction: *SHELXTL* (Version 6.1; Sheldrick, 2008), Fc^*^=kFc[1+0.001xFc^2^λ^3^/sin(2θ)]^-1/4^
Primary atom site location: structure-invariant direct methods	Extinction coefficient: 0.0086 (16)

### Special details

**Table:**

Geometry. All e.s.d.'s (except the e.s.d. in the dihedral angle between two l.s. planes) are estimated using the full covariance matrix. The cell e.s.d.'s are taken into account individually in the estimation of e.s.d.'s in distances, angles and torsion angles; correlations between e.s.d.'s in cell parameters are only used when they are defined by crystal symmetry. An approximate (isotropic) treatment of cell e.s.d.'s is used for estimating e.s.d.'s involving l.s. planes.
Refinement. Refinement of *F*^2^ against ALL reflections. The weighted *R*-factor *wR* and goodness of fit *S* are based on *F*^2^, conventional *R*-factors *R* are based on *F*, with *F* set to zero for negative *F*^2^. The threshold expression of *F*^2^ > σ(*F*^2^) is used only for calculating *R*-factors(gt) *etc*. and is not relevant to the choice of reflections for refinement. *R*-factors based on *F*^2^ are statistically about twice as large as those based on *F*, and *R*- factors based on ALL data will be even larger.

### Fractional atomic coordinates and isotropic or equivalent isotropic displacement parameters (Å^2^)

**Table:**

	*x*	*y*	*z*	*U*_iso_*/*U*_eq_	
O1A	0.6043 (2)	0.4234 (3)	0.45322 (7)	0.0357 (5)	
H1OA	0.632 (5)	0.537 (5)	0.4709 (13)	0.077 (12)*	
O2A	0.1728 (2)	0.2277 (2)	0.39933 (6)	0.0282 (4)	
O3A	0.2995 (2)	0.2761 (2)	0.48346 (7)	0.0330 (5)	
N1A	0.4517 (3)	0.3873 (3)	0.41951 (8)	0.0286 (5)	
C1A	0.4750 (3)	0.3877 (3)	0.36519 (10)	0.0284 (6)	
H2	0.3603	0.3618	0.3446	0.034*	
C2A	0.5993 (4)	0.5614 (4)	0.35413 (11)	0.0401 (7)	
H3	0.5380	0.6363	0.3455	0.048*	
H4	0.6908	0.6217	0.3840	0.048*	
C3A	0.6710 (4)	0.5081 (4)	0.30832 (11)	0.0417 (7)	
H3A	0.7319	0.5847	0.2864	0.050*	
C4A	0.6395 (4)	0.3422 (4)	0.30201 (11)	0.0396 (7)	
H4A	0.6735	0.2855	0.2745	0.047*	
C5A	0.5439 (3)	0.2528 (3)	0.34302 (10)	0.0297 (6)	
H5	0.6280	0.2422	0.3705	0.036*	
C6A	0.4023 (3)	0.0765 (3)	0.32410 (9)	0.0286 (6)	
C7A	0.3103 (4)	0.0433 (4)	0.27530 (10)	0.0326 (7)	
H6	0.3390	0.1303	0.2537	0.039*	
C8A	0.1752 (4)	−0.1155 (4)	0.25649 (10)	0.0363 (7)	
H7	0.1135	−0.1342	0.2227	0.044*	
C9A	0.1323 (4)	−0.2427 (4)	0.28649 (11)	0.0367 (7)	
H8	0.0412	−0.3500	0.2733	0.044*	
C10A	0.2221 (3)	−0.2172 (3)	0.33715 (10)	0.0306 (6)	
C11A	0.1782 (4)	−0.3471 (4)	0.36895 (11)	0.0372 (7)	
H11A	0.0884	−0.4553	0.3558	0.045*	
C12A	0.2619 (4)	−0.3208 (4)	0.41804 (12)	0.0406 (7)	
H12A	0.2302	−0.4096	0.4389	0.049*	
C13A	0.3959 (4)	−0.1609 (4)	0.43766 (11)	0.0374 (7)	
H13A	0.4550	−0.1427	0.4718	0.045*	
C14A	0.4416 (4)	−0.0323 (3)	0.40817 (10)	0.0312 (6)	
H14A	0.5312	0.0750	0.4223	0.037*	
C15A	0.3582 (3)	−0.0552 (3)	0.35665 (9)	0.0280 (6)	
C16A	0.3056 (3)	0.2920 (3)	0.43773 (10)	0.0268 (6)	
C17A	−0.0021 (3)	0.1253 (3)	0.40984 (9)	0.0295 (6)	
C18A	−0.1061 (4)	0.0807 (4)	0.35615 (10)	0.0396 (7)	
H18A	−0.0993	0.1866	0.3429	0.059*	
H18B	−0.2264	0.0099	0.3581	0.059*	
H18C	−0.0597	0.0167	0.3327	0.059*	
C19A	−0.0044 (4)	−0.0364 (3)	0.42982 (11)	0.0375 (7)	
H19A	0.0770	−0.0054	0.4619	0.056*	
H19B	0.0289	−0.1049	0.4037	0.056*	
H19C	−0.1204	−0.1036	0.4367	0.056*	
C20A	−0.0614 (4)	0.2358 (4)	0.44690 (11)	0.0369 (7)	
H20A	0.0147	0.2710	0.4802	0.055*	
H20B	−0.1794	0.1694	0.4524	0.055*	
H20C	−0.0576	0.3381	0.4319	0.055*	
O1B	0.4907 (3)	0.6687 (2)	0.05737 (8)	0.0385 (5)	
H1OB	0.557 (5)	0.667 (5)	0.0335 (13)	0.071 (12)*	
O2B	0.3481 (2)	0.2662 (2)	0.09741 (6)	0.0301 (4)	
O3B	0.3099 (2)	0.3398 (2)	0.01728 (7)	0.0352 (5)	
N1B	0.4828 (3)	0.5352 (3)	0.08597 (8)	0.0306 (5)	
C1B	0.5343 (3)	0.5915 (3)	0.14138 (10)	0.0296 (6)	
H10	0.5293	0.4885	0.1576	0.036*	
C2B	0.7184 (4)	0.7273 (4)	0.15626 (11)	0.0381 (7)	
H11	0.8036	0.6735	0.1596	0.046*	
H12	0.7483	0.8080	0.1307	0.046*	
C3B	0.7070 (4)	0.8157 (3)	0.20804 (11)	0.0380 (7)	
H3B	0.8040	0.8856	0.2328	0.046*	
C4B	0.5462 (4)	0.7841 (3)	0.21470 (11)	0.0369 (7)	
H13	0.5138	0.8284	0.2450	0.044*	
C5B	0.4195 (3)	0.6695 (3)	0.16871 (10)	0.0310 (6)	
H14	0.3883	0.7435	0.1457	0.037*	
C6B	0.2554 (3)	0.5356 (3)	0.18060 (10)	0.0276 (6)	
C7B	0.2584 (4)	0.4624 (4)	0.22474 (10)	0.0351 (7)	
H15	0.3642	0.4970	0.2472	0.042*	
C8B	0.1108 (4)	0.3382 (4)	0.23788 (11)	0.0394 (7)	
H16	0.1174	0.2898	0.2687	0.047*	
C9B	−0.0427 (4)	0.2871 (4)	0.20617 (11)	0.0404 (7)	
H17	−0.1429	0.2046	0.2155	0.049*	
C10B	−0.0543 (4)	0.3553 (3)	0.15974 (11)	0.0349 (7)	
C11B	−0.2113 (4)	0.2982 (4)	0.12554 (12)	0.0440 (8)	
H11B	−0.3122	0.2162	0.1348	0.053*	
C12B	−0.2198 (4)	0.3590 (4)	0.07978 (12)	0.0459 (8)	
H12B	−0.3258	0.3179	0.0571	0.055*	
C13B	−0.0730 (4)	0.4820 (4)	0.06591 (11)	0.0422 (8)	
H13B	−0.0803	0.5254	0.0341	0.051*	
C14B	0.0808 (4)	0.5398 (4)	0.09798 (10)	0.0330 (7)	
H14B	0.1796	0.6218	0.0877	0.040*	
C15B	0.0967 (3)	0.4806 (3)	0.14624 (10)	0.0292 (6)	
C16B	0.3713 (3)	0.3763 (3)	0.06313 (10)	0.0286 (6)	
C17B	0.2321 (4)	0.0831 (3)	0.08300 (10)	0.0312 (6)	
C18B	0.2446 (4)	0.0185 (4)	0.13454 (10)	0.0426 (8)	
H18D	0.3636	0.0327	0.1454	0.064*	
H18E	0.1692	−0.1034	0.1307	0.064*	
H18F	0.2091	0.0845	0.1608	0.064*	
C19B	0.0503 (4)	0.0681 (4)	0.06712 (11)	0.0376 (7)	
H19D	0.0130	0.1225	0.0958	0.056*	
H19E	−0.0263	−0.0535	0.0585	0.056*	
H19F	0.0463	0.1255	0.0367	0.056*	
C20B	0.2987 (4)	−0.0052 (4)	0.04207 (11)	0.0381 (7)	
H20D	0.4169	0.0097	0.0546	0.057*	
H20E	0.2969	0.0455	0.0102	0.057*	
H20F	0.2255	−0.1280	0.0349	0.057*	

### Atomic displacement parameters (Å^2^)

**Table:**

	*U*^11^	*U*^22^	*U*^33^	*U*^12^	*U*^13^	*U*^23^
O1A	0.0266 (11)	0.0377 (12)	0.0348 (11)	0.0093 (9)	−0.0081 (9)	−0.0052 (9)
O2A	0.0224 (10)	0.0306 (10)	0.0256 (9)	0.0043 (8)	0.0011 (8)	0.0027 (8)
O3A	0.0349 (12)	0.0330 (11)	0.0258 (10)	0.0084 (9)	0.0014 (8)	0.0020 (8)
N1A	0.0230 (13)	0.0302 (12)	0.0263 (11)	0.0061 (10)	−0.0029 (10)	−0.0014 (9)
C1A	0.0262 (15)	0.0256 (14)	0.0287 (14)	0.0057 (12)	0.0013 (12)	0.0023 (11)
C2A	0.0356 (18)	0.0331 (16)	0.0432 (17)	0.0037 (14)	0.0048 (14)	0.0074 (13)
C3A	0.0304 (17)	0.0429 (19)	0.0475 (18)	0.0056 (15)	0.0090 (14)	0.0191 (15)
C4A	0.0335 (17)	0.051 (2)	0.0355 (16)	0.0153 (15)	0.0111 (13)	0.0087 (14)
C5A	0.0280 (15)	0.0317 (15)	0.0286 (14)	0.0103 (13)	0.0044 (12)	0.0049 (12)
C6A	0.0297 (16)	0.0320 (15)	0.0264 (14)	0.0150 (13)	0.0065 (12)	0.0003 (12)
C7A	0.0386 (17)	0.0340 (16)	0.0282 (14)	0.0178 (14)	0.0053 (13)	0.0031 (12)
C8A	0.0387 (18)	0.0415 (18)	0.0286 (15)	0.0192 (15)	−0.0006 (13)	−0.0048 (13)
C9A	0.0325 (17)	0.0327 (16)	0.0392 (16)	0.0102 (14)	0.0029 (13)	−0.0074 (13)
C10A	0.0328 (16)	0.0265 (15)	0.0338 (15)	0.0138 (13)	0.0059 (13)	0.0002 (12)
C11A	0.0404 (18)	0.0241 (15)	0.0459 (18)	0.0119 (14)	0.0068 (15)	0.0020 (13)
C12A	0.049 (2)	0.0292 (16)	0.0482 (18)	0.0181 (15)	0.0119 (16)	0.0112 (14)
C13A	0.0421 (18)	0.0396 (17)	0.0337 (15)	0.0205 (15)	0.0026 (13)	0.0037 (13)
C14A	0.0332 (16)	0.0282 (15)	0.0318 (15)	0.0119 (13)	0.0033 (12)	0.0041 (12)
C15A	0.0291 (16)	0.0304 (15)	0.0282 (14)	0.0156 (13)	0.0068 (12)	0.0018 (12)
C16A	0.0265 (15)	0.0235 (14)	0.0276 (15)	0.0085 (12)	−0.0004 (12)	0.0004 (11)
C17A	0.0230 (15)	0.0308 (15)	0.0284 (14)	0.0042 (12)	0.0030 (12)	0.0020 (12)
C18A	0.0299 (17)	0.0461 (18)	0.0353 (16)	0.0081 (14)	0.0022 (13)	0.0021 (14)
C19A	0.0388 (18)	0.0296 (16)	0.0397 (16)	0.0078 (14)	0.0090 (14)	0.0035 (13)
C20A	0.0299 (16)	0.0363 (16)	0.0429 (16)	0.0115 (14)	0.0085 (13)	0.0013 (13)
O1B	0.0491 (14)	0.0319 (11)	0.0389 (11)	0.0159 (10)	0.0155 (10)	0.0143 (9)
O2B	0.0367 (11)	0.0238 (10)	0.0248 (9)	0.0076 (9)	−0.0009 (8)	0.0031 (8)
O3B	0.0370 (12)	0.0413 (12)	0.0261 (10)	0.0137 (10)	0.0046 (9)	0.0057 (8)
N1B	0.0369 (14)	0.0251 (12)	0.0304 (12)	0.0104 (11)	0.0089 (11)	0.0098 (10)
C1B	0.0288 (15)	0.0280 (15)	0.0300 (14)	0.0089 (13)	0.0053 (12)	0.0023 (12)
C2B	0.0279 (16)	0.0349 (16)	0.0458 (17)	0.0061 (13)	0.0072 (13)	0.0028 (14)
C3B	0.0324 (17)	0.0270 (15)	0.0453 (17)	0.0044 (13)	0.0003 (14)	−0.0021 (13)
C4B	0.0405 (19)	0.0273 (15)	0.0360 (16)	0.0086 (14)	0.0044 (14)	−0.0059 (12)
C5B	0.0291 (16)	0.0257 (15)	0.0374 (15)	0.0097 (13)	0.0050 (12)	0.0040 (12)
C6B	0.0276 (15)	0.0247 (14)	0.0309 (14)	0.0103 (12)	0.0077 (12)	0.0016 (11)
C7B	0.0342 (17)	0.0375 (17)	0.0350 (16)	0.0154 (14)	0.0082 (13)	0.0032 (13)
C8B	0.046 (2)	0.0403 (17)	0.0395 (16)	0.0207 (16)	0.0191 (15)	0.0131 (14)
C9B	0.0358 (18)	0.0336 (17)	0.0531 (19)	0.0122 (14)	0.0190 (16)	0.0054 (14)
C10B	0.0296 (17)	0.0305 (15)	0.0434 (17)	0.0112 (13)	0.0093 (14)	−0.0013 (13)
C11B	0.0298 (17)	0.0405 (18)	0.058 (2)	0.0112 (15)	0.0101 (15)	−0.0036 (16)
C12B	0.0288 (18)	0.053 (2)	0.054 (2)	0.0198 (16)	−0.0016 (15)	−0.0103 (17)
C13B	0.042 (2)	0.0512 (19)	0.0394 (17)	0.0276 (17)	0.0021 (15)	0.0004 (15)
C14B	0.0312 (16)	0.0338 (16)	0.0363 (16)	0.0154 (13)	0.0066 (13)	0.0031 (13)
C15B	0.0282 (16)	0.0256 (14)	0.0352 (15)	0.0126 (13)	0.0070 (12)	−0.0003 (12)
C16B	0.0302 (16)	0.0331 (16)	0.0249 (14)	0.0143 (13)	0.0068 (12)	0.0054 (12)
C17B	0.0356 (17)	0.0216 (14)	0.0306 (14)	0.0062 (13)	0.0033 (12)	−0.0006 (11)
C18B	0.057 (2)	0.0305 (16)	0.0341 (16)	0.0111 (15)	0.0031 (15)	0.0036 (13)
C19B	0.0315 (17)	0.0350 (16)	0.0384 (16)	0.0064 (14)	0.0063 (13)	−0.0050 (13)
C20B	0.0380 (18)	0.0313 (16)	0.0429 (16)	0.0133 (14)	0.0045 (14)	−0.0019 (13)

### Geometric parameters (Å, °)

**Table:**

O1A—N1A	1.405 (3)	O1B—N1B	1.407 (3)
O1A—H1OA	0.96 (4)	O1B—H1OB	0.88 (3)
O2A—C16A	1.337 (3)	O2B—C16B	1.337 (3)
O2A—C17A	1.480 (3)	O2B—C17B	1.479 (3)
O3A—C16A	1.223 (3)	O3B—C16B	1.222 (3)
N1A—C16A	1.363 (3)	N1B—C16B	1.357 (3)
N1A—C1A	1.454 (3)	N1B—C1B	1.450 (3)
C1A—C2A	1.534 (4)	C1B—C2B	1.536 (4)
C1A—C5A	1.554 (3)	C1B—C5B	1.553 (3)
C1A—H2	1.0000	C1B—H10	1.0000
C2A—C3A	1.497 (4)	C2B—C3B	1.509 (4)
C2A—H3	0.9900	C2B—H11	0.9900
C2A—H4	0.9900	C2B—H12	0.9900
C3A—C4A	1.321 (4)	C3B—C4B	1.319 (4)
C3A—H3A	0.9500	C3B—H3B	0.9500
C4A—C5A	1.503 (4)	C4B—C5B	1.510 (4)
C4A—H4A	0.9500	C4B—H13	0.9500
C5A—C6A	1.521 (4)	C5B—C6B	1.515 (4)
C5A—H5	1.0000	C5B—H14	1.0000
C6A—C7A	1.375 (4)	C6B—C7B	1.371 (4)
C6A—C15A	1.436 (4)	C6B—C15B	1.431 (4)
C7A—C8A	1.404 (4)	C7B—C8B	1.402 (4)
C7A—H6	0.9500	C7B—H15	0.9500
C8A—C9A	1.361 (4)	C8B—C9B	1.366 (4)
C8A—H7	0.9500	C8B—H16	0.9500
C9A—C10A	1.418 (4)	C9B—C10B	1.410 (4)
C9A—H8	0.9500	C9B—H17	0.9500
C10A—C11A	1.411 (4)	C10B—C11B	1.417 (4)
C10A—C15A	1.427 (4)	C10B—C15B	1.428 (4)
C11A—C12A	1.362 (4)	C11B—C12B	1.359 (4)
C11A—H11A	0.9500	C11B—H11B	0.9500
C12A—C13A	1.409 (4)	C12B—C13B	1.400 (4)
C12A—H12A	0.9500	C12B—H12B	0.9500
C13A—C14A	1.362 (4)	C13B—C14B	1.369 (4)
C13A—H13A	0.9500	C13B—H13B	0.9500
C14A—C15A	1.421 (4)	C14B—C15B	1.421 (4)
C14A—H14A	0.9500	C14B—H14B	0.9500
C17A—C19A	1.513 (4)	C17B—C19B	1.511 (4)
C17A—C18A	1.517 (4)	C17B—C20B	1.514 (3)
C17A—C20A	1.526 (3)	C17B—C18B	1.518 (4)
C18A—H18A	0.9800	C18B—H18D	0.9800
C18A—H18B	0.9800	C18B—H18E	0.9800
C18A—H18C	0.9800	C18B—H18F	0.9800
C19A—H19A	0.9800	C19B—H19D	0.9800
C19A—H19B	0.9800	C19B—H19E	0.9800
C19A—H19C	0.9800	C19B—H19F	0.9800
C20A—H20A	0.9800	C20B—H20D	0.9800
C20A—H20B	0.9800	C20B—H20E	0.9800
C20A—H20C	0.9800	C20B—H20F	0.9800
			
N1A—O1A—H1OA	104 (2)	N1B—O1B—H1OB	107 (2)
C16A—O2A—C17A	120.66 (19)	C16B—O2B—C17B	121.51 (19)
C16A—N1A—O1A	114.3 (2)	C16B—N1B—O1B	115.0 (2)
C16A—N1A—C1A	125.8 (2)	C16B—N1B—C1B	125.7 (2)
O1A—N1A—C1A	113.7 (2)	O1B—N1B—C1B	113.83 (19)
N1A—C1A—C2A	113.2 (2)	N1B—C1B—C2B	114.0 (2)
N1A—C1A—C5A	114.6 (2)	N1B—C1B—C5B	115.4 (2)
C2A—C1A—C5A	105.8 (2)	C2B—C1B—C5B	105.2 (2)
N1A—C1A—H2	107.7	N1B—C1B—H10	107.3
C2A—C1A—H2	107.7	C2B—C1B—H10	107.3
C5A—C1A—H2	107.7	C5B—C1B—H10	107.3
C3A—C2A—C1A	101.8 (2)	C3B—C2B—C1B	101.4 (2)
C3A—C2A—H3	111.4	C3B—C2B—H11	111.5
C1A—C2A—H3	111.4	C1B—C2B—H11	111.5
C3A—C2A—H4	111.4	C3B—C2B—H12	111.5
C1A—C2A—H4	111.4	C1B—C2B—H12	111.5
H3—C2A—H4	109.3	H11—C2B—H12	109.3
C4A—C3A—C2A	112.1 (3)	C4B—C3B—C2B	111.8 (3)
C4A—C3A—H3A	124.0	C4B—C3B—H3B	124.1
C2A—C3A—H3A	124.0	C2B—C3B—H3B	124.1
C3A—C4A—C5A	112.6 (2)	C3B—C4B—C5B	112.3 (2)
C3A—C4A—H4A	123.7	C3B—C4B—H13	123.8
C5A—C4A—H4A	123.7	C5B—C4B—H13	123.8
C4A—C5A—C6A	115.7 (2)	C4B—C5B—C6B	116.6 (2)
C4A—C5A—C1A	100.6 (2)	C4B—C5B—C1B	100.6 (2)
C6A—C5A—C1A	113.1 (2)	C6B—C5B—C1B	113.5 (2)
C4A—C5A—H5	109.0	C4B—C5B—H14	108.6
C6A—C5A—H5	109.0	C6B—C5B—H14	108.6
C1A—C5A—H5	109.0	C1B—C5B—H14	108.6
C7A—C6A—C15A	118.9 (2)	C7B—C6B—C15B	118.8 (3)
C7A—C6A—C5A	119.6 (2)	C7B—C6B—C5B	119.6 (2)
C15A—C6A—C5A	121.5 (2)	C15B—C6B—C5B	121.6 (2)
C6A—C7A—C8A	121.9 (3)	C6B—C7B—C8B	122.3 (3)
C6A—C7A—H6	119.1	C6B—C7B—H15	118.9
C8A—C7A—H6	119.1	C8B—C7B—H15	118.9
C9A—C8A—C7A	120.2 (3)	C9B—C8B—C7B	119.8 (3)
C9A—C8A—H7	119.9	C9B—C8B—H16	120.1
C7A—C8A—H7	119.9	C7B—C8B—H16	120.1
C8A—C9A—C10A	120.8 (3)	C8B—C9B—C10B	120.9 (3)
C8A—C9A—H8	119.6	C8B—C9B—H17	119.5
C10A—C9A—H8	119.6	C10B—C9B—H17	119.5
C11A—C10A—C9A	121.6 (3)	C9B—C10B—C11B	121.4 (3)
C11A—C10A—C15A	119.3 (2)	C9B—C10B—C15B	119.2 (3)
C9A—C10A—C15A	119.1 (2)	C11B—C10B—C15B	119.4 (3)
C12A—C11A—C10A	121.4 (3)	C12B—C11B—C10B	121.0 (3)
C12A—C11A—H11A	119.3	C12B—C11B—H11B	119.5
C10A—C11A—H11A	119.3	C10B—C11B—H11B	119.5
C11A—C12A—C13A	119.6 (3)	C11B—C12B—C13B	120.3 (3)
C11A—C12A—H12A	120.2	C11B—C12B—H12B	119.8
C13A—C12A—H12A	120.2	C13B—C12B—H12B	119.8
C14A—C13A—C12A	120.7 (3)	C14B—C13B—C12B	120.2 (3)
C14A—C13A—H13A	119.7	C14B—C13B—H13B	119.9
C12A—C13A—H13A	119.7	C12B—C13B—H13B	119.9
C13A—C14A—C15A	121.4 (3)	C13B—C14B—C15B	121.8 (3)
C13A—C14A—H14A	119.3	C13B—C14B—H14B	119.1
C15A—C14A—H14A	119.3	C15B—C14B—H14B	119.1
C14A—C15A—C10A	117.6 (2)	C14B—C15B—C10B	117.2 (3)
C14A—C15A—C6A	123.3 (2)	C14B—C15B—C6B	123.6 (3)
C10A—C15A—C6A	119.1 (2)	C10B—C15B—C6B	119.1 (2)
O3A—C16A—O2A	126.1 (2)	O3B—C16B—O2B	125.9 (2)
O3A—C16A—N1A	123.3 (2)	O3B—C16B—N1B	124.1 (2)
O2A—C16A—N1A	110.4 (2)	O2B—C16B—N1B	109.9 (2)
O2A—C17A—C19A	109.8 (2)	O2B—C17B—C19B	109.9 (2)
O2A—C17A—C18A	101.86 (19)	O2B—C17B—C20B	110.5 (2)
C19A—C17A—C18A	110.2 (2)	C19B—C17B—C20B	113.5 (2)
O2A—C17A—C20A	109.7 (2)	O2B—C17B—C18B	100.9 (2)
C19A—C17A—C20A	113.1 (2)	C19B—C17B—C18B	109.6 (2)
C18A—C17A—C20A	111.5 (2)	C20B—C17B—C18B	111.6 (2)
C17A—C18A—H18A	109.5	C17B—C18B—H18D	109.5
C17A—C18A—H18B	109.5	C17B—C18B—H18E	109.5
H18A—C18A—H18B	109.5	H18D—C18B—H18E	109.5
C17A—C18A—H18C	109.5	C17B—C18B—H18F	109.5
H18A—C18A—H18C	109.5	H18D—C18B—H18F	109.5
H18B—C18A—H18C	109.5	H18E—C18B—H18F	109.5
C17A—C19A—H19A	109.5	C17B—C19B—H19D	109.5
C17A—C19A—H19B	109.5	C17B—C19B—H19E	109.5
H19A—C19A—H19B	109.5	H19D—C19B—H19E	109.5
C17A—C19A—H19C	109.5	C17B—C19B—H19F	109.5
H19A—C19A—H19C	109.5	H19D—C19B—H19F	109.5
H19B—C19A—H19C	109.5	H19E—C19B—H19F	109.5
C17A—C20A—H20A	109.5	C17B—C20B—H20D	109.5
C17A—C20A—H20B	109.5	C17B—C20B—H20E	109.5
H20A—C20A—H20B	109.5	H20D—C20B—H20E	109.5
C17A—C20A—H20C	109.5	C17B—C20B—H20F	109.5
H20A—C20A—H20C	109.5	H20D—C20B—H20F	109.5
H20B—C20A—H20C	109.5	H20E—C20B—H20F	109.5

### Hydrogen-bond geometry (Å, °)

**Table:**

*D*—H···*A*	*D*—H	H···*A*	*D*···*A*	*D*—H···*A*
O1A—H1OA···O3A^i^	0.96 (4)	1.75 (4)	2.689 (3)	165 (3)
O1B—H1OB···O3B^ii^	0.88 (3)	1.84 (4)	2.714 (3)	179 (4)

Symmetry codes: (i) −*x*+1, −*y*+1, −*z*+1; (ii) −*x*+1, −*y*+1, −*z*.
